# *Haemophilus influenzae* Invasive Infections in Children in Vaccine Era: Phenotypic and Genotypic Characterization Tunis, Tunisia

**DOI:** 10.3390/microorganisms12122666

**Published:** 2024-12-23

**Authors:** Yasmine Chelbi, Khaoula Meftah, Ala-Eddine Deghmane, Samar Mhimdi, Firas Aloui, Aida Bouafsoun, Eva Hong, Khaled Menif, Khadija Boussetta, Monia Khemiri, Samir Boukthir, Mehdi Trifa, Said Jlidi, Riadh Jouini, Zohra Fitouri, Mohamed-Nabil Nessib, Muhamed-Kheir Taha, Hanen Smaoui

**Affiliations:** 1 Laboratory of Microbiology, Children’s Hospital of Tunis, Beb Saadoun, Tunis 1007, Tunisia; meftahkhaoula@gmail.com (K.M.); samarmem@gmail.com (S.M.); faloui571@gmail.com (F.A.); aidabouaf@gmail.com (A.B.); 2 Faculty of Medicine, University of Tunis El Manar, Beb Saadoun, Tunis 1007, Tunisia; menifk@yahoo.fr (K.M.); khedija.boussetta@rns.tn (K.B.); monia.khemiri@rns.tn (M.K.); samir.boukthir@rns.tn (S.B.); mehditrifa@gmail.com (M.T.); said853@rns.tn (S.J.); jouiniriadh@yahoo.fr (R.J.); zohra.fitouri@rns.tn (Z.F.); nabil.nessib@rns.tn (M.-N.N.); 3 Invasive Bacterial Infections Unit, National Reference Centre for Meningococci and Haemophilus Influenza, Institut Pasteur, CEDEX 15 Paris, France; ala-eddine.deghmane@pasteur.fr (A.-E.D.); eva.hong@pasteur.fr (E.H.); muhamed-kheir.taha@pasteur.fr (M.-K.T.); 4 Department of Pediatric Intensive Care, Children’s Hospital of Tunis, Beb Saadoun, Tunis 1007, Tunisia; 5 Department of Paediatrics B, Children’s Hospital of Tunis, Beb Saadoun, Tunis1007, Tunisia; 6 Department of Paediatrics A, Children’s Hospital of Tunis, Beb Saadoun, Tunis 1007, Tunisia; 7 Department of Paediatrics C, Children’s Hospital of Tunis, Beb Saadoun, Tunis 1007, Tunisia; 8 Department of Anaesthesia and Intensive Care, Children’s Hospital of Tunis, Beb Saadoun, Tunis 1007, Tunisia; 9 Department of Paediatric Surgery B, Children’s Hospital of Tunis, Beb Saadoun, Tunis 1007, Tunisia; 10 Department of Paediatric Surgery A, Children’s Hospital of Tunis, Beb Saadoun, Tunis 1007, Tunisia; 11 Department of Paediatrics D, Children’s Hospital of Tunis, Beb Saadoun, Tunis 1007, Tunisia; 12 Department of Pediatric Orhtopedic Surgery, Children’s Hospital of Tunis, Beb Saadoun, Tunis 1007, Tunisia

**Keywords:** *Haemophilus influenzae*, invasive infections, child, serotyping, drug resistance bacterial, molecular typing

## Abstract

The changing epidemiological profile of invasive *Haemophilus influenzae* infections (IIHi) is noted in the post-vaccination era. The aim of this study was to characterize phenotypically and genotypically invasive *Haemophilus influenzae* (Hi) isolates detected in Tunisian pediatric patients. A retrospective study was conducted in the microbiology laboratory of the Children’s Hospital of Tunis over ten years (2013–2023). All IIHi cases were included. Molecular identification and serotyping were conducted through qPCR. Molecular typing and analysis of resistance genes were extracted from whole genome sequencing data. Fifty-three IIHi cases were collected. Children under five years old were the most affected (81%). Non-typable isolates (NTHi) were predominant (79%) followed by serotype b (17%) and serotype a (4%). Genetic diversity was observed, essentially among NTHi isolates. Resistance of Hi isolates to ampicillin, amoxicillin–clavulanic acid and cefotaxime (CTX) were 42%, 20% and 4%, respectively. Thirteen isolates (29%) produced a beta-lactamase and 14 carried the *blaTEM-1* gene (kappa = 0.95). For non-enzymatic resistance, group 3 (n = 12) showed resistance to ampicillin. Groupe 4 (n = 9, NTHi) showed discordances with resistance to CTX. The emergence of resistance to CTX is concerning. Continuous surveillance through molecular tools in conjunction with phenotypic and clinical data is necessary to ensure better management of these infections.

## 1. Introduction

*Haemophilus influenzae* (Hi), a Gram-negative coccobacillus, is a natural commensal of the human upper respiratory tract [1]. Indeed, Hi disease includes respiratory or ear, nose and throat (ENT) infections as well as severe invasive infections (IIHi), mainly in children under five years old, such as meningitis, bacteremic pneumonia, bacteriemia and septic arthritis [2]. Hi can be surrounded by a polysaccharide capsule belonging to one of the six distinct capsular serotypes (a through f), with serotype b (Hib) being the most virulent. Non-capsulated isolates can also be encountered and are named non-typeable (NT) Hi (NTHi). A considerable shift in the epidemiological pattern of IIHi has been observed since the introduction of the type b Hi vaccine (Hib) in 1985, with an incidence rate regression of more than 90% [3]. Hib, which was the primary serotype implicated in IIHi before vaccination (95%), has become rare [3]. However, a recent increase in IIHi has been reported in many areas [4,5]. NTHi isolates have become predominant in Europe and South Africa (78% and 64%, respectively) [3]. A slight increase in serotypes e and f has also been observed in Europe [3,4]. Moreover, an emergence of serotype a (Hia) has been observed in specific populations [6,7,8]. Moreover, Hib invasive infections have been reported as increasing recently in France and the Netherlands [9,10]. Furthermore, a change in Hi’s antibiotic susceptibility profile has been observed in the post-vaccination period [11]. Two main mechanisms can lead to beta-lactam resistance: enzymatic, involving production of beta-lactamase, and non-enzymatic, involving the modification of penicillin-binding protein 3 (PBP3) through mutation in the encoding gene, *ftsI*. A rise in the non-enzymatic mechanism has been noted in recent years [11]. Moreover, additional mutations in *ftsI* that lead to isolates that are resistant to third-generation cephalosporins (TGC), which constitute first-line treatment for IIHi, have also been detected worldwide [12,13,14]. In Tunisia, the Hib vaccine was introduced into the national vaccination schedule in 2002, withdrawn in 2005 due to budgetary reasons and then reintroduced in April 2011. It consists of three doses, administered to infants at two, three and six months of age, with no booster dose. All IIHi cases, primarily meningitis, were, before Hib vaccine introduction, due to serotype b [15]. This bacterium represented the leading cause of bacterial meningitis in children in Tunisia before vaccination, with an incidence rate of 16 to 25 cases per 100,000 children under five years old [16]. Resistance to amoxicillin due to beta-lactamase production was 37.2%, while non-enzymatic resistance to amoxicillin remained low (1.6%) [15]. Limited data exist about IIHi in the post-vaccination era in Tunisia, particularly in children [17]. The aim of this study was to characterize phenotypically and genotypically invasive Hi isolates detected in Tunisian pediatric patients after the implementation of Hib vaccination.

## 2. Materials and Methods

### 2.1. Type of Study and Bacterial Isolates

A retrospective study was conducted in the microbiology laboratory of Bechir Hamza Children’s Hospital of Tunis (BHCH) over ten years between 1 May 2013 and 30 April 2023. All IIHi cases were included. IIHi was defined as the detection of Hi in sterile sites (blood, cerebrospinal fluid (CSF), synovial fluid (SF) or pleural fluid (PF)), either by culture and/or by polymerase chain reaction (PCR) [18]. Samples were collected from patients under the age of 14 admitted to BHCH during the study period or to other Tunisian hospitals that requested laboratory confirmation of invasive bacterial infection (IBI) by PCR between June 2019 and April 2023, which was performed at the BHCH within the framework of the “Multicenter Surveillance Study of IBI in North Africa” (MENINGSTOP) [2]. Redundant isolates from the same patient and the same infectious episode were excluded. In this study, the sampling criteria for pediatric patients remained consistent throughout and corresponded to the detection of Hi at a sterile site. All samples were received in sterile tubes or in BactAlert (Biomérieux ^®^, Craponne, France) blood culture bottles and processed according to the same laboratory protocol.

Demographic and clinical data about the study population were obtained according to two pre-established forms, filled according to medical records or accompanying samples sent as part of MENINGSTOP project [2]. Data concerning antimicrobial susceptibility testing were extracted using the SIR scan system (i2a diagnostic^®^, Paris, France).

### 2.2. Identification and Serotyping

Phenotypic identification of Hi isolates was based on morphologic and cultural characteristics as well as the requirement for V (NAD) and X (hemin) factors using factor-impregnated disks (Diatabs^TM^ Rosco Diagnostica, Copenhagen, Denmark).

Deoxyribonucleic acid (DNA) extraction of Hi isolates, stored at −80 °C in brain–heart infusion broth supplemented with 15% glycerol, were performed using the heat shock method [19]. For culture-negative samples with positive PCR (CNSPP), bacterial DNA extraction was performed using an automated technique on EZ1 (Qiagen^®^, Hilden, Germany) or MagNA pure (Roche^®^, Pleasanton, CA, USA).

Molecular identification and serotyping were carried out through two real-time PCR (RT-PCR) using Taqman technology, according to the World Health Organization (WHO) protocol and to Michel et al. [20,21]. They consist of a first multiplex RT-PCR targeting the *hpd* gene for identification and the *bexD* gene for determining the encapsulated nature of the Hi isolate and then a second simplex RT-PCR targeting *acs* and *bcs* genes encoding the specific proteins for serotypes a and b. Amplification was conducted on the LightCycler^®^ 480II thermocycler (Roche^®^, Pleasanton, CA, USA).

### 2.3. Antimicrobial Susceptibility Testing

Disk diffusion was performed using penicillin G (PG) (1 µg), rifampicin (RIF) (5 µg), trimethoprim–sulfamethoxazole (SXT) (1.25–23.75 µg), chloramphenicol (CHL) (30 µg), and tetracycline (TET) (30 µg) disks according to Antibiogram Committee of the French Society of Microbiology—European Committee on Antimicrobial Susceptibility Testing (CA-SFM/EUCAST) recommendations (https://www.sfm-microbiologie.org/ (accessed on 8 January 2024)). Minimal inhibitory concentrations (MIC) of ampicillin (AMP), amoxicillin clavulanic acid (AMC), cefotaxime (CTX), meropenem (MEM) and ciprofloxacin (CIP) were determined for viable isolates using E-Test Strips (Biomérieux^®^, Craponne, France). Available inhibition diameters as well as retested antibiotics MICs were interpreted according to the CA-SFM/EUCAST 2023 guidelines. Beta-lactamase production was detected using an acidimetric test (Beta-lactamase test DiatabsTM Rosco Diagnostica, Copenhagen, Denmark) according to the manufacturer’s guidelines for isolates that show an inhibition zone diameter around the PG disk less than 12 mm.

Isolates were classified according to AMP MIC and production of beta-lactamase into three groups: beta-lactamase negative AMP susceptible (BLNAS) (MICAMP ≤ 1 mg/L), beta-lactamase positive AMP resistant (BLPAR) and beta-lactamase negative AMP resistant (BLNAR).

Antibiograms were regularly controlled using Hi strain ATCC 49247 (American Type Culture Collection, Manassas, VA, USA).

### 2.4. Whole Genome Sequencing

WGS was conducted at the Institut Pasteur Paris, France, in collaboration with the Invasive Bacterial Infections Unit and the National Reference Center for Meningococci and *Haemophilus influenzae* (NRCMHi). Extraction of genomic DNA was performed using the MagNa Pure 96 kit (Roche Molecular Systems, Pleasanton, CA, USA). Libraries were prepared with the Nextera XT DNA Library Preparation kit (Illumina, San Diego, CA, USA). The WGS technology used was Illumina (NextSeq^®^ 500, Illumina, USA). Genomes were assembled using SPAdes software (CAB, Saint Petersburg State University, Saint Petersburg, Russia). Sequences obtained are available at PubMLST (https://pubmlst.org/ (accessed on 4 February 2024)).

Phylogenetic trees were established and visualized using SplitsTree4 (version 4.14.6). WGS data were used for confirming bacterial identification and conducting molecular typing using the available tools on www.pubmlst.org. such as species identification (by rMLST) and allelic analysis including antibiotic resistance genes.

The presence or absence of *blaTEM-1* and *blaROB-1* encoding for Hi beta-lactamase was studied using the BLAST tool. *ftsI* gene mutations were detected by analyzing a 621 bp fragment using BIGSdb platform. Sequence alignment was performed using Multiple Sequence Alignment CLUSTALW (available on https://www.genome.jp/tools-bin/clustalw (accessed on 23 April 2024)). A specific *ftsI* allele number was assigned to isolates with at least one nucleotide difference using the PubMLST database. A phylogenetic tree based on amino acid sequences deduced from *ftsI* alleles was also constructed via Splitstree4. These alleles were categorized into four groups based on phylogenetic tree clustering, considering the presence or absence of critical mutations conferring resistance to AMP and/or CTX according to literature data [18]. In fact, group 1 and group 2 were known to be correlated to a susceptible phenotype to beta-lactams, group 3 to resistance to amoxicillin but not to cefotaxime and group 4 to resistance to cefotaxime. Mutations in *gyrA* and *parC* genes responsible for resistance to fluoroquinolone, as well as *folP* and *rpoB* genes, which confer resistance to SXT and RIF, respectively, were also screened. The presence of *cat* gene encoding enzymatic resistance to CHL was also screened using blast tool on pubmlst.org.

A molecular typing of Hi isolates through a Multi-Locus Sequence Typing (MLST) analysis, extracted from whole genome sequencing (WGS) results, was conducted.

### 2.5. Statistical Analysis

Data were analyzed using IBM SPSS statistics version 26 (IBM Corp., Armonk, NY, USA). Qualitative variables were compared using the chi-square test or Fisher’s exact test. Concordance between phenotypic antibiotic susceptibility testing and resistance gene studying was assessed using Kappa coefficient. A *p*-value ≤ 0.05 was considered statistically significant.

### 2.6. Ethical Considerations

The protocol of this study was approved by the local ethics committee of HEBHT under number 03/2024. All samples studied were collected for routine diagnostic purposes of IIHi. Patient data were processed anonymously.

## 3. Results

### 3.1. Clinico-Epidemiological Characteristics of Study Population

**Figure 1:**
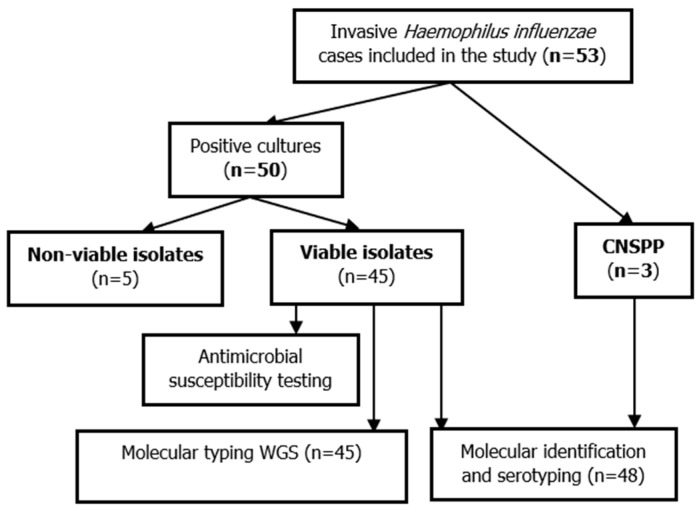
Flowchart of the study. WGS: Whole genome sequencing; CNSPP: culture-negative samples, positive PCR.

In the study period, 53 IIHi cases were included; three were only confirmed by PCR (CNSPP) (n = 3 CSF) and 50 by culture (Figure 1). Among IIHi cases with positive cultures, five isolates failed to be resuscitated (Figure 1). Isolates were detected in 35 blood cultures (66%), 14 CSF (26%), two PF (4%), one SF (2%) and one bone sample (2%). IIHi cases predominantly occurred in children under five years old (n = 43, 81%), mainly those under six months (n = 30, 57%) (Table 1). The median age was 5.1 months, with a range from one day to 10 years, and the sex ratio was 1.4.

Clinical data were available for 46 patients. IIHi cases presented as bacteremic pneumonia (n = 26, 57%), meningitis (n = 14, 31%), pleuropneumonia (n = 2, 4%), maternal–fetal infection (bacteremia) (n = 2, 4%), septic arthritis (n = 1, 2%) and osteomyelitis (n = 1, 2%). Meningitis cases were more frequent in patients aged over six months (*p* = 0.01), and bacteremic pneumonia cases were more frequent in those aged under six months (*p* = 0.012).

IIHi cases showed an inflammatory syndrome, including a median CRP level of 77 mg/L [Q1 = 30; Q3 = 150] and a median white blood cell count of 15,000/mm^3^ [Q1 = 11,500; Q3 = 20,800]. CSF’s cytological analysis revealed a median protein level of 1.1 g/L [Q1 = 0.47; Q3 = 1.75], a median CSF/blood glucose ratio of 0.4% [Q1 = 0.34; Q3 = 0.45], a median white blood cell count of 744 cells/mm3 [Q1 = 300; Q3 = 1970] and a median percentage of neutrophilic polynuclear cells of 80% [Q1 = 80; Q3 = 90].

Complications were observed in 9 patients (20%). Invasive mechanical ventilation or vasopressor therapy was needed in 20 (44%) and five (11%) patients, respectively. Only one death was recorded, in a 9-month-old infant with bacteremic pneumonia, who had no previous medical history.

### 3.2. Identification, Serotyping and Molecular Typing

All cultured isolates were phenotypically identified. Molecular identification and serotyping included 45 viable isolates and 3 CNSPP. Only one isolate from a blood culture was *hpd*-negative. rMLST extracted from WGS confirmed that all 45 isolates belonged to Hi species. WGS analysis revealed that the *hpd*-negative isolate harbored a truncated 5′ end of the *hpd* gene in this strain, explaining the absence of annealing of the forward primer.

The majority of IIHi cases resulted from non-encapsulated isolates (79%, 38/48) (Table 1). Most encapsulated isolates were primarily serotype b (17%). The two Hia isolates exhibited mucoid colony aspects in culture and were isolated from CSF in patients aged under six months.

Blood cultures were predominantly positive for NTHi isolates (94%, 29/31), while CSF were more frequently positive for encapsulated isolates (7/13) (*p* = 0.002) (Table 1). NTHi isolates were as likely to lead to complications as encapsulated isolates (*p* = 0.31). Data about vaccination were available for 6/8 patients with Hib invasive disease. In fact, Hib cases were diagnosed in one non-vaccinated and three incompletely vaccinated patients. Two cases occurred in completely vaccinated and non-immunocompromised patients. The first patient was 17 months old and had poly-malformities and a history of prematurity, while the second was five years old and had no medical history.

**Figure 2:**
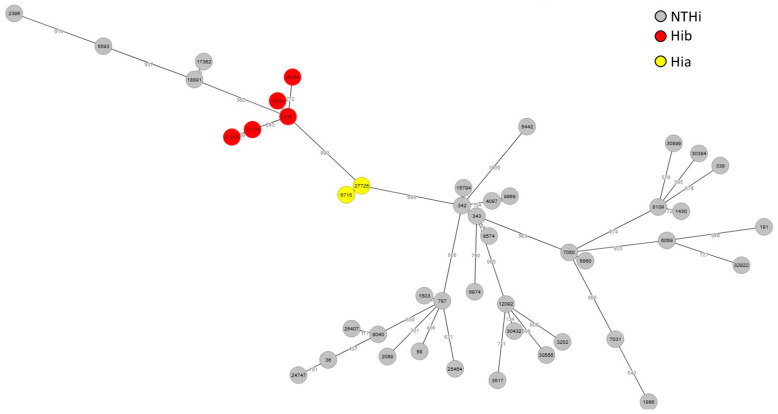
GrapeTree based on allelic profile of the cgMLST loci of invasive *Haemophilus influenzae* isolates. Isolates were indicated as in the Supplementary Table S1 and were colored according to their serotypes. The number between the neighboring isolates indicated the number of different alleles of genes included in the cgMLST scheme (numbers refer to isolates references); encapsulated isolates were homogenously grouped compared to non-typeable *Haemophilus influenzae* isolates.

Molecular typing using WGS of the 45 viable isolates showed 20 clonal complexes (CC). Clonal complexes ST-57 (n = 6/45, 13%) and ST-6 (n = 5, 11%) were predominant. Six isolates were unclassified. For NTHi isolates, a high genetic diversity was observed with CC ST-57, which accounted for 16% (6/38), and CC ST-107, which accounted for 8% (3/38). Encapsulated isolates were more homogenously grouped, with Hia isolates belonging to the same CC ST-23 (n = 2) and Hib isolates to CC ST-6 (n = 5) (Figure 2).

### 3.3. Antibiotic Susceptibility Testing

The study of beta-lactam resistance profiles in invasive Hi isolates indicated elevated resistance levels for AMP (42%, 19/45), with 20% resistance to AMC (9/45) and 29% (13/45) isolates producing beta-lactamase. All isolates were susceptible to MEM.

Classification based on the beta-lactam resistance groups revealed 26/45 BLNAS (58%), 13/45 BLPAR (29%) and 6/45 BLNAR (13%). All BLNAS isolates were sensitive to CTX (MICmax = 0.125 mg/L) (Table 2). For BLPAR isolates, 3/13 were also resistant to AMC through *ftsI* mutations (belonging to group 4 of *ftsI*), of which two (4%) isolates were also resistant to CTX with CTX MICs of 1.5 and 256 mg/L. These isolates were from blood cultures and were multidrug-resistant, with resistance also observed against SXT, TET and CHL. The six BLNAR isolates harbored *ftsI* mutations were all resistant to AMC with four isolates in group 3 and two isolates in group 4.

Distribution of beta-lactam resistance according to serotypes revealed that all BLNAR isolates were NTHi (Table 1). Both Hia isolates were classified as BLNAS, whereas Hib isolates were either BLPAR or BLNAS (Table 1).

Molecular screening for beta-lactamase genes revealed that 14/45 isolates (31%) were harboring the *blaTEM-1* gene. The *blaROB-1* gene was not detected. An excellent agreement was found between phenotypic and molecular screening for beta-lactamase with a kappa coefficient of 0.95 (*p* < 0.0001). Only one isolate carried *blaTEM-1* (allele 27) despite a negative beta-lactamase test. This allele showed a polymorphism in the promoter -35 region in the corresponding isolate.

**Figure 3:**
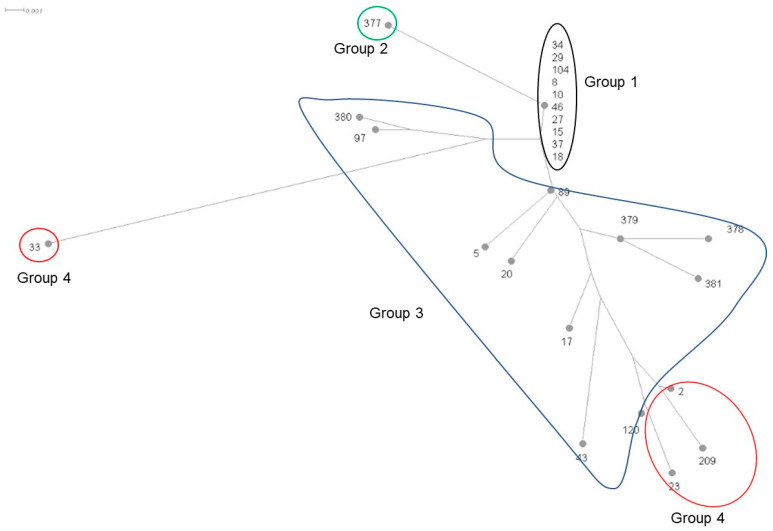
Phylogenetic tree of the *ftsI* based on the sequence CLUSTALW multiple alignment of amino-acid sequences deduced of the DNA sequences of all *ftsI* alleles defined among the isolates of this study (Numbers refer to *ftsI* alleles); Group 1 and Group 2 were known to be correlated with a susceptible phenotype to beta-lactams, Group 3 to resistance to amoxicillin but not to cefotaxime and Group 4 to resistance to cefotaxime; these alleles are grouped homogenously on the phylogenetic tree according to their *ftsI* group.

Regarding molecular profile of the *ftsI* gene, 26 distinct alleles were identified, including five new alleles that were assigned (Figure 3). Allele 8 was predominant (16%, 7/45), followed by allele 10 (11%, 5/45) and allele 2 (11%, 5/45). In this study, group 1 included alleles 8, 10, 15, 18, 27, 29, 34, 37, 46 and 104. Group 2 included one new allele, *ftsI* 377, with no critical mutations. Twenty-four isolates (53%) were related to these two groups; ten (42%) harbored the beta-lactamase encoding gene (including the isolate with the non-detectable production of beta-lactamase), and nine isolates were classified as BLPAR (Figure 3 and Table 2). The remaining isolates were classified as BLNAS. The MIC of AMP for non-beta-lactamase-producing isolates ranged from 0.19 to 0.5 mg/L.

Twenty-one (47%) isolates were affiliated with groups 3 and 4 and consequently exhibited critical mutations in *ftsI*. All isolates were classified as NTHi, which seems to be associated with the presence of critical mutations in *ftsI* (*p* = 0.01).

Group 3 included alleles 5, 17, 20, 43, 89, 97 and 120 and four novel alleles (378, 379, 380 and 381). Corresponding isolates (n = 12) carried the critical mutation N526K and/or R517H isolated or associated with other mutations. The MIC of AMP of non-beta-lactamase-producing isolates belonging to this group ranged from 0.38 to 1.5 mg/L. Isolates were classified as 7 BLNAS, 4 BLNAR, and 1 BLPAR. No resistance to CTX was observed in this group. BLNAR isolates carried alleles 17, 20, 43, and 89.

Group 4 included alleles 2, 23, 33 and 209. It was represented by 9 isolates, of which 4 were BLNAS, 3 were BLPAR and 2 were BLNAR. All isolates exhibited critical mutations M3771 and D350N associated with other mutations. Only two isolates were resistant to CTX in our study. They carried allele 2 and mutations D350N, M377I, A502V and N526K. These same mutations were found in four other isolates classified as either BLPAR (n = 1), BLNAR (n = 1) or BLNAS (n = 2) but with a positive screening for beta-lactam resistance by PG disc. A low concordance was obtained between isolates resistant to CTX and its belonging to group 4 (kappa = 0.31; *p* = 0.004).

Regarding susceptibility profile to fluoroquinolone, 2/45 (4%) isolates were resistant to CIP with MICs of 1.5 and 32 mg/L. These isolates were classified as BLPAR and BLNAR respectively. WGS results revealed the presence of the S84L mutation in the *gyrA* gene and the S84I mutation in the *parC* gene for the first isolate, and the presence of the S84L and D88G mutations in the *gyrA* gene, associated with the S84I mutation in the *parC* gene, for the second isolate.

Resistance to SXT was reported in 16 (36%) isolates. Half of the AMP-resistant Hi isolates were susceptible to SXT (9/19). *folP* gene sequencing showed some discrepancies between SXT resistance profile and absence of critical mutations for four isolates. However, a very good agreement with a kappa of 0.8 (*p* < 0.0001) was still found.

Concerning other antibiotics, the three CHL-resistant isolates (6%) were found to harbor the *cat* gene. Only one isolate (2%) was resistant to RIF, but no mutations in the *rpoB* gene were detected. Resistance to TET was observed in 5 isolates (11%).

## 4. Discussion

This study of IIHi in children from BHCH of Tunis allowed us to determine the epidemiological profile of these severe infections in the post-vaccination era and to describe the resistance profile, implicated serotypes and molecular diversity of Hi isolates. The majority of IIHi in this study occurred in children under five years of age. Similar results were found in the United States [22]. A slight male predominance was observed, in agreement with other studies [18].

Hi was mainly isolated in blood cultures and CSF, consistent with NRCMHi data in 2017 [18].

Meningitis was more likely to be caused by encapsulated isolates as reported by authors from Texas children’s hospital in a study between 2011 and 2018 [22]. Complications related to IIHi were not rare, occurring in 20% of cases, which was similar to a Japanese study (23.1%) [23]. Complications were due to NTHi as much as encapsulated Hi isolates highlighting the need for particular attention to these non-encapsulated strains. The only recorded death in this study was due to an NTHi isolate. Studies demonstrated higher case–fatality ratios associated with IIHi caused by non-encapsulated isolates compared to encapsulated non-b Hi (3.8% to 9.7% versus 2% to 4.7%, respectively) [24]. However, Hib was related to a higher case–fatality ratio particularly in neonates (10%) [24].

The detection of *hpd*-negative isolates suggests that amplifying two genes simultaneously would be interesting to increase diagnostic accuracy of these severe infections. Indeed, targeting the *fucK* or *omp2* genes has demonstrated sensitivity and specificity rates of 97.1% and 100%, respectively [25].

A predominance of NTHi has been observed in many regions since the widespread use of the Hib conjugate vaccine, which was also noted in our study [3]. In Europe (2018) and the United States (2021), NTHi caused 78% and 67.4% of IIHi, respectively [4,5]. NTHi is responsible for the majority of IIHi in all age groups and mainly affects newborns, young children, pregnant women and the elderly, particularly in patients with underlying comorbidities and impaired immunity [3,26]. The exact reasons behind this propensity, however, remain unclear. The introduction of a NTHi vaccine seems to be worth considering particularly for high-risk groups [27,28]. Indeed, implementing a large-scale NTHi vaccine to prevent invasive disease does not appear to be cost-effective due to low incidence [29]. Some NTHi vaccines are currently under investigation [30]. Serotype b accounted for 17% of IIHi in our study, while a study in the pre-vaccine era demonstrated that all invasive Hi isolates in Tunis were serotype b [15]. The rate of Hib invasive infections was higher than that observed in Europe (7%) and the Unites States (3%) [4,5]. Some studies demonstrated that vaccination schedule consisting of three doses without a booster, as the case in Tunisia, may lead to declining antibody levels over time [31,32]. Additionally, Heath et al. demonstrated that 44% of vaccinated infants with vaccine failure who presented with IIHi had risk factors such as prematurity, congenital malformations or neutropenia [33]. Indeed, among two completely vaccinated patients with invasive Hib infections in our study, one had a history of prematurity and malformations.

Two Hia isolates (4%) were detected in this study. This serotype primarily affects indigenous populations in the United States, Canada and Australia, and data suggest its introduction in Europe [6,7,8,34]. Our rates were comparable with those observed in a French study including 501 invasive isolates (4%) [9]. In terms of virulence, serotype a ranked second after serotype b [35]. Its virulence may be explained by presence of adhesion factors and hyperproduction of the capsule [36]. Indeed, despite their susceptibility to beta-lactams, both Hia isolates exhibited a mucoid aspect in culture and were responsible for severe clinical presentations. A conjugate Hia vaccine is being studied and appears promising for vulnerable populations mainly among autochthons in the USA and Canada [34,37]. Hia invasive infections mainly affected infants under six months, consistent with literature data [24].

Molecular typing revealed a high genetic diversity among Hi isolates causing invasive infections in Tunisian children. This diversity primarily involved NTHi. Indeed, this diversity is reported in the literature and is due to the tendency of non-encapsulated strains to undergo mutations and recombinations [38]. Non-encapsulated isolates belonging to CC ST-103 and ST-160 were found to be more associated with invasive infections [38]. However, only one isolate was classified as ST-103 in our study. A French study showed that 96% of Hib isolates belonged to CC ST 6, known to be highly associated with invasive infections, which was consistent with our findings [9]. Regarding Hia, ST 23 is predominant in France, which was also observed in our study [18]. A British study showed that invasive strains were mainly ST-1511 (39%), ST-23 (25%) and ST-56 (14%) [37]. These results emphasize the importance of expanding the use of molecular typing to better understand the virulence of Hi clones.

Beta-lactams are the preferred treatment for IIHi. However, resistance to AMP is steadily increasing worldwide, leading to therapeutic challenges [39]. In France, 23% and 10% of isolates causing IIHi between 2017 and 2021 were resistant to AMP and AMC, respectively [14]. Our study noted higher rates, possibly attributable to increased antibiotic consumption in our region. Resistance to CTX concerned 4% of the isolates, which is similar to resistance rates in France (3%) [14]. Resistance to CTX is rare. Indeed, a Spanish study did not detect any isolates resistant to third-generation cephalosporin over 12 years [38]. In Germany, the CTX resistance rate was 0.9% between 2016 and 2019 [12]. A global emergence of multidrug-resistant and extensively drug-resistant strains reaching 26.6% was reported [39].

Beta-lactam resistance is mediated with two mechanisms of resistance. The first mechanism of resistance is enzymatic, through production of TEM-1 or ROB-1 beta-lactamases. Isolates carrying *blaTEM-1* are more widespread; however, *blaROB-1* has been essentially detected in the United States, Canada and Mexico [40]. The enzymatic mechanism was implicated in 16% of beta-lactam resistance in France between 2017 and 2021 [14]. Our study showed higher rates, reaching 29%. A good agreement between phenotypic expression of beta-lactamase and presence of the *blaTEM-1* gene was noted (kappa = 0.95; *p* < 0.0001). These results aligned with a Norwegian study that demonstrated 100% sensitivity, specificity, negative and positive predictive values of molecular resistance markers to detect AMP resistance in isolates with no PBP3 mediated resistance [13]. The non-detection of beta-lactamase production for one isolate in our study may be explained by observed polymorphism in the promoter -35 region, which may be responsible for a lower production of beta-lactamase [41]. A Chinese study suggested that polymorphism in the promoter region could lead to variable levels of resistance to beta-lactams [42]. Pdel-type promoters could thus be associated with susceptibility to AMP.

The second mechanism of resistance to beta-lactams is non-enzymatic, through *ftsI* gene mutations. All BLNAR strains in our study were non-encapsulated. This predominance of BLNAR in NTHi isolates was also described by Deghmane et al. [18]. Typeable isolates are known to be more susceptible to beta-lactams, particularly Hia, for which resistance remains infrequent [6,43].

WGS analysis revealed high diversity of the *ftsI* gene, with 26 different alleles identified. A classification of these alleles into four groups was proposed by Deghmane et al. [18]. In our study, MICs for AMP in non-beta-lactamase producing isolates from group 1 and 2 were consistent with the genotypic resistance study. Group 3 isolates harbored critical mutations associated with resistance to AMP, which aligned with AMP MICs observed [43]. Regarding Group 4, only 2/9 isolates, harboring critical mutations conferring resistance to CTX, showed phenotypic concordance (kappa = 0.31). Deghmane et al. found an excellent concordance between CTX resistance and Group 4 classification, which may be explained by the lower frequency of mutations S385T and L389F in our study [18] (Supplementary Material Table S1). It has been demonstrated that these mutations, particularly L389F, confer resistance to CTX and are associated with high CTX MICs [12]. The variable impacts of Group 4 mutations on the phenotype of susceptibility/resistance to beta-lactams (including CTX) may also be linked to the 3-dimensional structure of the whole PBP3 and its interaction with beta-lactam antibiotics. This aspect needs further exploration as alleles of group 4 seem to have arisen from alleles of group 3 at separate events as suggested by the phylogenetic tree (Figure 3). Moreover, further investigations are needed to better understand the role of each mutation in the non-enzymatic beta-lactam resistance.

Detection of fluoroquinolone resistance in invasive Hi isolates was noted in our study, but the prevalence remains low. A combination of two *gyrA* mutations, particularly a D88G mutation, along with a *parC* mutation, is reported to confer high-level resistance to fluoroquinolone, with MICs that exceed 32 mg/L [44]. Fluoroquinolone use in pediatric patients should however be conducted carefully to prevent adverse effects and antimicrobial resistance [45]. Indeed, the American Academy of Pediatrics recommends limiting fluoroquinolone administration to multidrug-resistant infections without other alternatives. Additionally, it is interesting to note that half of the AMP-resistant Hi isolates were susceptible to SXT in this study. Authors suggest its potential use as first-line treatment in non-invasive infections such as acute otitis media [43]. This antibiotic usefulness in invasive infections has yet to be determined.

## 5. Conclusions

Data from this study suggest a considerable shift in the serotype distribution of IIHi in the post-vaccination era in Tunisia, with a predominance of NTHi isolates and the emergence of Hia isolates. Although for pneumococcal infections, a substitution of vaccine serotypes with non-vaccine ones was noted, serotype replacement remains controversial for Hi [34]. Moreover, Hib invasive infections seem to be re-increasing in several European countries in spite of high vaccination coverage and the overall cases due to Hia isolates, for instance, remain low [9,10,18].

High diversity, resistance to beta-lactams and the potential morbimortality of these isolates emphasize the need to better investigate their virulence mechanism and to define new vaccination targets. A vaccine targeting non-encapsulated Hi and/or a bivalent Hib and Hia vaccine would be very beneficial in reducing the morbimortality of IIHi. The main limitations of this study are its single-center design, which restricts our ability to generalize results to the entire pediatric population in Tunisia, and its retrospective nature leading to the loss of some results. Establishing a national surveillance network, particularly molecular surveillance through NGS sequencing platforms, appears essential for gaining better insight into IIHi pattern in Tunisia and for further exploring the role of *ftsI* mutations in conferring Hi resistance to beta-lactams. Continuous surveillance is mandatory to detect any potential serotype replacement and to revise vaccination strategies.

## Figures and Tables

**Table 1:** Sample sites, clinical characteristics and antimicrobial susceptibility testing of invasive *Haemophilus influenzae* cases according to serotype.

Serotype		NTHi	Hib	Hia	Total
Sample site	CSF	6 (46%)	5 (39%)	2 (15%)	13
	BC	29 (94%)	2 (6%)	-	31
	Other	3 (75%)	1 (25%)	-	4
Age	<6 M	21 (78%)	4 (15%)	2 (7%)	27
	[6 M–5 Y]	11 (85%)	2 (15%)	-	13
	>5 Y	3 (60%)	2 (40%)	-	5
Clinical characteristics	Meningitis	6 (46%)	5 (39%)	2 (15%)	13
	Pneumonia	22 (92%)	2 (8%)	-	24
	Other	4 (80%)	1 (20%)	-	5
	Complications	5 ^a^ (63%)	1 ^b^ (13%)	2 ^c^ (24%)	8
	Deaths	1 (100%)	-	-	1
Vaccination status	Complete	6 (75%)	2 (25%)	-	8
	Incomplete	3 (38%)	3 (38%)	2 (24%)	8
	Not vaccinated	15 (94%)	1 (6%)	-	16
Antimicrobial susceptibility testing	BLNAS	21 (81%)	3 (11%)	2 (8%)	26
	BLPAR	11 (85%)	2 (15%)	-	13
	BLNAR	6 (100%)	-	-	6

^a^ Septic shock, acute respiratory distress syndrome, syndrome of inappropriate antidiuretic hormone; ^b^ cardiorespiratory arrest; ^c^ empyema and septic shock. NTHi: non typable *Haemophilus influenzae*; Hib: *Haemophilus influenzae* serotype b; Hia: *Haemophilus influenzae* serotype a; BLNAS: betalactamase-negative, ampicillin susceptible; BLPAR: betalactamase-positive, ampicillin resistant; BLNAR: betalactamase-negative, ampicillin resistant; CSF: cerebrospinal fluid; BC: blood culture; M: months; Y: years.

**Table 2:** Minimal inhibitory concentrations, categorization to beta-lactams and *ftsI* groups of invasive *Haemophilus influenzae* isolates.

		MIC50 ^a^	MIC90 ^a^	Min ^a^	Max ^a^	S ^b^	R ^b^	*ftsI* Group		
								1 + 2	3	4
All (n = 45)	AMP	1	256	0.19	256	26	19	24	12	9
	AMC	1.5	128	0.38	256	36	9			
	CTX	0.023	0.125	0.008	32	43	2			
BLNAS (n = 26)	AMP	0.38	1	0.19	1	26	0	15	7	4
	AMC	0.75	1.5	0.38	1.5	26	0			
	CTX	0.016	0.094	0.008	0.125	26	0			
AMP-R (n = 19)	AMP	64	256	1.5	256	0	19	9	5	5
	AMC	2	256	0.75	256	10	9			
	CTX	0.032	1.5	0.008	32	17	2			
BLPAR (n = 13)	AMP	192	256	1.5	256	0	13	9	1	3
	AMC	1.5	256	0.75	256	10	3			
	CTX	0.023	24	0.008	32	11	2			
BLNAR (n = 6)	AMP	1.5	2	1.5	2	0	6	0	4	2
	AMC	8	256	3	256	0	6			
	CTX	0.055	0.125	0.047	0.125	6	0			

^a^ mg/L ^b^ (n=) AMP: ampicillin; AMC: amoxicillin clavulanic acid; CTX: cefotaxime; R: resistant; S: susceptible; Min: minimum; Max: maximum; BLNAS: beta-lactamase-negative, ampicillin susceptible; BLPAR: beta-lactamase-positive, ampicillin resistant; BLNAR: beta-lactamase-negative, ampicillin resistant.

## Data Availability

The authors declare that the data supporting the findings of this study are available within the paper and its Supplementary Information file. Should any raw data files be needed in another format, they are available from the corresponding author upon reasonable request.

## Supplementary Materials

The following supporting information can be downloaded at: https://www.mdpi.com/article/10.3390/microorganisms12122666/s1, Table S1: Serotypes, beta-lactam resistance profile and *ftsI* mutations of *Haemophilus influenzae* isolates (n = 45).
