# Supplementary material for: Haemophilus influenzae Invasive Infections in Children in Vaccine Era: Phenotypic and Genotypic Characterization Tunis, Tunisia
Source: Microorganisms. 2024 Dec 23;12(12):2666. doi: 10.3390/microorganisms12122666 (PMC11728474; doi:10.3390/microorganisms12122666)
Supplement: Supplementary file 1 [file microorganisms-12-02666-s001.zip › microorganisms-3300945-supplementary.pdf]

Haemophilus influenzae invasive infections in children in vaccine era: Phenotypic and genotypic characterization Tunis, Tunisia

(Supplementary material)

Table S1: Serotypes, beta-lactams resistance profile and *ftsI* mutations of *Haemophilus influenzae* isolates (n=45)

Ref	Year	Age	Site	Serotype	PG	MIC AMP	MIC AMC	MIC CTX	BL Group	*TEM-1*	*ftsI*	*ftsI* mutations*
**32100**	2013	5 m	BC	Hib	R	256	0.75	0,008	BLPAR	+	Group 1	WT
**5593**	2014	7 m	BC	NTHi	S	0.25	0.75	0,008	BLNAS	-	Group 1	WT
**18891**	2016	3 m	BC	NTHi	S	0.25	0.5	0,008	BLNAS	+	Group 1	WT
**27725**	2016	5 m	CSF	Hia	S	0.5	0.5	0.016	BLNAS	-	Group 1	WT
**30556**	2016	9 m	BC	NTHi	R	256	1.5	0.016	BLPAR	+	Group 1	WT
**191**	2017	3 m	BC	NTHi	S	0.38	0.75	0.008	BLNAS	-	Group 1	WT
**8109**	2017	NP	PF	NTHi	S	0.25	0.5	0.008	BLNAS	-	Group 1	WT
**27905**	2017	2 y	CSF	Hib	S	0.25	0.75	0.012	BLNAS	-	Group 1	WT
**30899**	2017	2 y	BC	NTHi	S	0.25	0.75	0.012	BLNAS	-	Group 1	WT
**32367**	2017	8 y	AF	Hib	S	0.25	0.75	0.008	BLNAS	-	Group 1	WT
**7050**	2018	3 m	BC	NTHi	R	256	1.5	0.023	BLPAR	+	Group 1	WT

**8574**	2019	US	CSF	NTHi	S	0.38	1	0.016	BLNAS	-	Group 1	WT
**36**	2020	2 m	BC	NTHi	R	12	1.5	0.032	BLPAR	+	Group 1	WT
**6059**	2020	6 y	CSF	NTHi	R	8	1.5	0.023	BLPAR	+	Group 1	WT
**5860**	2021	2 y	CSF	NTHi	R	64	1.5	0.016	BLPAR	+	Group 1	WT
**6715**	2021	4 m	CSF	Hia	S	0.125	0.38	0.008	BLNAS	-	Group 1	WT
**18084**	2021	1 y	BC	Hib	S	0.25	0.5	0.012	BLNAS	-	Group 1	WT
**25064**	2021	5 y	CSF	Hib	R	256	1.5	0.008	BLPAR	+	Group 1	WT
**4097**	2022	18 d	BC	NTHi	S	0.38	0.75	0.012	BLNAS	-	Group 1	WT
**9869**	2022	1 y	BC	NTHi	S	0.25	0.75	0.016	BLNAS	-	Group 1	WT
**32922**	2023	8 y	BC	NTHi	S	0.25	0.75	0.023	BLNAS	-	Group 1	WT
**3517**	2023	4 d	BC	NTHi	R	1.5	1.5	0.023	BLPAR	+	Group 1	WT
**6974**	2023	10 y	CSF	NTHi	R	256	2	0.032	BLPAR	+	Group 1	WT
**1986**	2018	1 m	BC	NTHi	S	0.38	0.75	0.012	BLNAS	-	Group 2	New allele
**5442**	2015	17 d	BC	NTHi	R	1.5	12	0.064	BLNAR	-	Group 3	D350N; A502V; N526K
**6787**	2016	1 m	BC	NTHi	S	1	1.5	0.047	BLNAS	-	Group 3	D350N; G490E; N526K
**2396**	2016	2 y	BC	NTHi	R	0.75	1.5	0.023	BLNAS	-	Group 3	A502T; N526K
**30394**	2017	1 m	BC	NTHi	S	0.75	1	0.016	BLNAS	-	Group 3	A502V; N526k

**30432**	2017	9 m	BC	NTHi	S	1	1.5	0.032	BLNAS	-	Group 3	D350N; G490E; A502V; N526K
**26407**	2018	4 d	BC	NTHi	R	1.5	256	0.125	BLNAR	-	Group 3	N526K
**1603**	2019	4 d	BC	NTHi	R	1.5	256	0.094	BLNAR	-	Group 3	D350N; G490E; N526K
**24747**	2021	18 d	BC	NTHi	R	256	2	0.023	BLPAR	+	Group 3	A502V; N526K
**58**	2022	1 y	Bone	NTHi	S	0.38	0.75	0.012	BLNAS	-	Group 3	A502V; R517H
**2089**	2022	9 d	BC	NTHi	R	1.5	3	0.047	BLNAR	-	Group 3	N526K
**12092**	2022	1 m	BC	NTHi	R	0.75	1.5	0.064	BLNAS	-	Group 3	A502T; N526k
**17362**	2022	1 y	CSF	NTHi	S	0.75	1	0.016	BLNAS	-	Group 3	I449V; N526K
**343**	2016	2 m	BC	NTHi	R	256	256	1.5	BLPAR	+	Group 4	D350N; M377I; A502V; N526K
**3202**	2016	2 m	BC	NTHi	R	2	3	0.046	BLNAR	-	Group 4	D350N; M377I; A502V; R517H; N526K
**25464**	2018	5 m	BC	NTHi	R	1.5	3	0.047	BLNAR	-	Group 4	D350N; M377I; G490E; A502V
**339**	2019	US	BC	NTHi	R	0.5	1.5	0.032	BLNAS	-	Group 4	D350N; M377I; A502V; N526K
**342**	2020	2 m	BC	NTHi	R	256	256	32	BLPAR	+	Group 4	D350N; M377I; A502V; N526K

**1430**	2021	5 m	BC	NTHi	R	0.25	0.38	0.023	BLNAS	-	Group 4	D350N; M377I; A502V; N526K
**15794**	2021	1 y	BC	NTHi	R	256	3	0.047	BLPAR	+	Group 4	D350N; M377I; A502V; N526K
**7031**	2023	1 y	CSF	NTHi	R	1	1.5	0.094	BLNAS	-	Group 4	D350N; M377I; G490E; A502V
**9040**	2023	5 m	PF	NTHi	R	1	1.5	0.125	BLNAS	-	Group 4	D350N; S357N; M377I; S385T; R517H

*Blue and red colors refer to isolates belonging to group 3 and 4 respectively

Ref : reference ; y: years ; m: months ; d: days ; PG : penicillin G ; MIC : minimal inhibitory concentration ; AMP : ampicillin ; AMC : amoxicillin-clavulanic acid ; CTX : cefotaxime ; BL : beta-lactamine ; BC : blood culture ; CSF : cerebrospinal fluid ; PF : pleural fluid ; AF : articulair fluid ; US : unspecified ; S : sensible ; R : résistant ; BLPAR : beta-lactamase positive ampicillin resistant ; BLNAR : beta-lactamase negative ampicillin resistant ; BLNAS : beta-lactamase negative ampicillin suceptible ; WT : wild type; Hia *: Haemophilus influenzae* a ; Hib : *Haemophilus influenzae* b ; NTHi : Non typeable *Haemophilus influenzae*
